# Trivial Excitation Energy Transfer to Carotenoids Is an Unlikely Mechanism for Non-photochemical Quenching in LHCII

**DOI:** 10.3389/fpls.2021.797373

**Published:** 2022-01-13

**Authors:** Callum Gray, Tiejun Wei, Tomáš Polívka, Vangelis Daskalakis, Christopher D. P. Duffy

**Affiliations:** ^1^Digital Environment Research Institute (DERI), Queen Mary University of London, London, United Kingdom; ^2^Department of Physics, Faculty of Science, University of South Bohemia, Ceske Budejovice, Czechia; ^3^Department of Chemical Engineering, Cyprus University of Technology, Limassol, Cyprus

**Keywords:** carotenoid, non-photochemical quenching (NPQ), LHCII, energy-dissipation, photosystem (PSII), transient absorption

## Abstract

Higher plants defend themselves from bursts of intense light via the mechanism of Non-Photochemical Quenching (NPQ). It involves the Photosystem II (PSII) antenna protein (LHCII) adopting a conformation that favors excitation quenching. In recent years several structural models have suggested that quenching proceeds via energy transfer to the optically forbidden and short-lived *S*_1_ states of a carotenoid. It was proposed that this pathway was controlled by subtle changes in the relative orientation of a small number of pigments. However, quantum chemical calculations of *S*_1_ properties are not trivial and therefore its energy, oscillator strength and lifetime are treated as rather loose parameters. Moreover, the models were based either on a single LHCII crystal structure or Molecular Dynamics (MD) trajectories about a single minimum. Here we try and address these limitations by parameterizing the vibronic structure and relaxation dynamics of lutein in terms of observable quantities, namely its linear absorption (LA), transient absorption (TA) and two-photon excitation (TPE) spectra. We also analyze a number of minima taken from an exhaustive meta-dynamical search of the LHCII free energy surface. We show that trivial, Coulomb-mediated energy transfer to *S*_1_ is an unlikely quenching mechanism, with pigment movements insufficiently pronounced to switch the system between quenched and unquenched states. Modulation of *S*_1_ energy level as a quenching switch is similarly unlikely. Moreover, the quenching predicted by previous models is possibly an artifact of quantum chemical over-estimation of *S*_1_ oscillator strength and the real mechanism likely involves short-range interaction and/or non-trivial inter-molecular states.

## 1. Introduction

Non-photochemical quenching (NPQ) in higher plants is a regulatory response to a sudden increase in light intensity (Horton et al., 2000; Niyogi, 2000; Müller et al., 2001; Ruban et al., 2012). It is a (mostly Malnoë et al., 2018) reversible down-regulation of the quantum efficiency of the Photosystem II (PSII) light-harvesting antenna (LHCII) with the purpose of defending the saturated reaction centers from over-excitation and photoinhibition (Powles, 1984; Aro et al., 1993). Essentially, it is due to the creation of exciton-quenching species within LHCII which trap and dissipate chlorophyll excitation before it can accumulate in PSII and damage the reaction centers. While the fine molecular details of the mechanism are still unclear, a general consensus has emerged over the basic scheme. The primary trigger of NPQ is an acidification of the thylakoid lumen (ΔpH) due to a high rate of electron transport (Strand and Kramer, 2014), in large part arising from cyclic electron flow about PSI (Sato et al., 2014). The ΔpH activates three components of NPQ: the PSII antenna sub-unit PsbS (Li et al., 2004), the enzyme violaxanthin de-epoxidase (VDE) (Jahns et al., 2009), and the LHCII antenna proteins themselves (Walters et al., 1994; Liu et al., 2008; Belgio et al., 2013). VDE converts the violaxanthin pool to zeaxanthin which may lead to violaxanthin-zeaxanthin exchange in the loose, peripheral xanthophyll-binding site of LHCII (Xu et al., 2015). It has been shown that the presence of zeaxanthin affects the kinetics and amplitude of NPQ but is not a strict requirement for it (Ruban and Horton, 1999; Nicol and Croce, 2021). Quenching can similarly be achieved in the absence of PsbS if ΔpH is driven to non-physiological levels (Johnson and Ruban, 2011). Either way (for further information, the reader is directed to a comprehensive review of this complex and on-going topic Ruban, 2016) the combined effect is to induce an in-membrane aggregation or clustering of LHCII (Horton et al., 1991) and some subtle internal conformational changes (Ilioaia et al., 2011). These somehow modulate the pigment-pigment and pigment-protein couplings to create a quenching species, although the nature of the quencher and molecular dynamics of the conformational “switch” are still unclear.

Recently, it has become broadly (though by no means universally) accepted that the quencher is or involves one of the LHCII carotenoid (Cart) pigments (Ma et al., 2003). These are attractive candidates as they are, in a sense, intrinsically quenched, possessing a very short (≈10 ps) excitation lifetime relative to chlorophyll (Chl – ≈4–6 ns). Various mechanisms have been suggested, such as excitation energy transfer (EET) to the Cart which quenches simply by virtue of its short lifetime (Ruban et al., 2007); mixing of the Chl and Cart lifetimes brought on by excitonic resonance (Bode et al., 2009; Holleboom and Walla, 2014); and formation of fast-relaxing Chl-Cart CT states (Holt et al., 2005; Ahn et al., 2008). The lutein (Lut) bound to the L1 binding site (Wei et al., 2016) of the LHCII trimer is often cited (Ruban et al., 2007) as being the particular carotenoid involved in quenching, but zeaxanthin at an equivalent site in one of the minor PSII antennae has also been proposed (Ahn et al., 2008). We also note that Holzwarth and co-workers present a Cart-independent quencher model that involves Chl-Chl CT states (Müller et al., 2010; Ostroumov et al., 2020). The differences between these models often come down to specific interpretations of highly-congested time-resolved spectral measurements on these complexes. Moreover, any involvement of the Carts is obscured by the fact that their lowest singlet excitation, *S*_1_, is optically forbidden and decays very quickly (Polívka and Sundström, 2004).

The X-ray structure of LHCII (Liu et al., 2004) can provide some insight into the quencher, particularly since it was found to correspond to a highly dissipative configuration, meaning it could serve as a model structure for the quenched state (Pascal et al., 2005). Several detailed models of this structure very accurately predicted the steady state and time-resolved spectra of LHCII (Novoderezhkin et al., 2004, 2011; Müh et al., 2010) but they did not capture the dissipative character (in fairness that was never their goal). One possible reason for this was their neglect of the Carts, due to the fact that they contribute nothing to the spectrum in the red region and that there are no truly reliable methods for calculating the excitation energy and one-electron transition density of the *S*_1_ state. The latter is due to the strong electron correlations giving it a complex multi-electron character (Tavan and Schulten, 1987; Andreussi et al., 1993). Beginning in 2013 Duffy and co-workers used a semi-empirical quantum chemistry method to estimate the *S*_1_ transition density and its potential effect on the excitation lifetime of the LHCII crystal structure (Duffy et al., 2013; Chmeliov et al., 2015; Fox et al., 2017, 2018). These models suggested that quenching was due to EET from the Chl *Q*_*y*_ band to the *S*_1_ state of the centrally bound Luts (L1 and L2), followed by fast decay of *S*_1_. This EET was mediated by weak resonance couplings between *Q*_*y*_ and *S*_1_ (due to the latter's lack of oscillator strength) and was therefore assumed to be incoherent (Förster transfer) and slow (20–50 ps) relative to excitation equilibration across the Chls (≈1–2 ps). This is essentially the mechanism proposed by Ruban et al. based on global target analysis of transient absorption (TA) measurements on LHCII aggregate, although they propose L1 as the sole quencher (Ruban et al., 2007). Of course these models are all based on a single, time-averaged structural configuration, and a highly artificial one at that. It therefore tells us nothing about how such quenching is switched on and off and can only very tentatively be applied to the actual *in vivo* quenching mechanism. More recently, the model was extended to a molecular dynamics simulation of the LHCII trimer within a lipid bilayer (Balevičius et al., 2017). Although a stable, unquenched conformation was not identified, it predicted that the *Q*_*y*_ − *S*_1_ coupling was highly sensitive to very small changes in inter-pigment orientations, suggesting that the lifetime could be modulated by very subtle conformational changes. Unfortunately, this appears to have been incorrect for two reasons. Firstly, the coupling sensitivity appears to have been an artifact of the semi-empirical Hamiltonian used to calculate *S*_1_. Khokhlov and Belov showed that this sensitivity disappears when more rigorous methods are used (Khokhlov and Belov, 2019). Moreover, by simulating the near-identical CP29, Lapillo et al. showed that even with the semi-empirical method, large lifetime fluctuations are significantly dampened if one accounts for the excitonic structure of the Chl manifold in the complex (the previous model assumed a Chl-Lut dimer embedded in some coarse-grained, iso-energetic Chl pool) (Lapillo et al., 2020). In addition to these errors, the model has a series of weaknesses that here we attempt to address:

- ***The S***_**1**_ ***excitation energy*** is neither easy to measure directly or calculate. Transient absorption in near-IR gives a phononless excitation energy of 14,050 ± 300cm^−1^ for Lut in recombinant LHCII (Polívka et al., 2002), while two-photon excitation (TPE) in native LHCII gave < 15,300cm^−1^ (Walla et al., 2000). The latter value is likely the first vibronic peak which is ≈1100cm^−1^ higher than the phononless peak, meaning the two values agree reasonably well. Nevertheless, it is often treated as a free parameter and large changes to its value have been proposed as a part of the NPQ switch (Holleboom and Walla, 2014; Lapillo et al., 2020).
- ***The vibronic structure and relaxation dynamics of S***_**1**_ were not properly considered. It was treated as a single optical transition with a line-broadening function chosen to provide a convincing visual fit to the TPE spectrum which implied very large reorganization energies. It was assumed that reorganization on *S*_1_ was instantaneous and that internal conversion (IC) to the ground state (*S*_0_) occurred with a single rate constant of ≈ 10−20ps (Polívka et al., 2002). The end result is a picture of *S*_1_ as an deep, irreversible trap. In reality, *S*_1_ is composed of several vibronic transitions that could couple differently to *Q*_*y*_, relax on finite timescales and undergo IC at different rates.
- ***Limited sampling of the LHCII potential energy surface (PES)*** means we might not be probing biologically relevant conformations. Unsteered MD simulations start from a quenched minimum close to the crystal structure. Single molecule spectroscopy has shown that LHCII trimers will spontaneously switch between quenched and unquenched states but the typical dwell time in each is of the order 1–10 s (Krüger et al., 2010), meaning prohibitively long unsteered simulations may be needed to capture this switching.

Here we attempt to correct these errors in several ways. We obtain a detailed picture of the *S*_1_ energy gap, vibronic structure and relaxation kinetics by fitting a detailed model to the TA kinetics of Lut in pyridine. These parameters (along with a secular Redfield model of the Chl manifold Malý et al., 2016) are then used to model energy relaxation in LHCII. The LHCII model structures that we use come from an exhaustive steered search of the LHCII PES which was previously published (Daskalakis et al., 2020). The motivation is to determine whether NPQ can realistically be switched on and off simply by altering the relative distance/orientation of Lut and its neighboring chlorophylls.

## 2. Results

### 2.1. Steady-State Spectra of the Chlorophyll Excitonic Manifold

**Figure 1:**
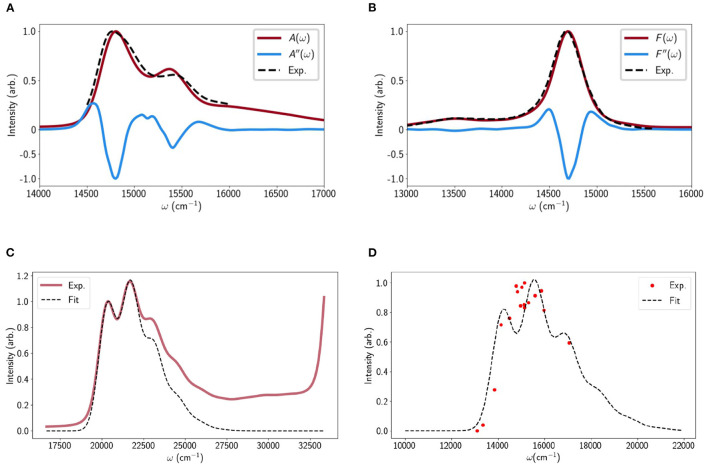
**(A)** Calculated linear absorption spectrum of LHCII derived from one of the minima (red line) compared to the experimental spectrum (dashed line) (Krüger et al., 2010). The second derivative of the calculated absorption is shown (blue line) to highlight the Chl a and b peaks. The calculated spectra were essentially identical for all LHCII minima probed. **(B)** Calculated and experimental fluorescence profiles. **(C)** The calculated (dashed line) and measured linear absorption spectra of Lut in pyridine. **(D)** The calculated (dashed line) and measured (red dots) (Walla et al., 2002) TPE spectrum of Lut. All calculation parameters were taken from the TA fit apart from a 150cm^−1^ blue-shift to account for the fact that the TPE measurements were performed in octanol.

The Chl-Chl relaxation dynamics are modeled according to the method in Malý et al. (2016) and briefly recapped in the Methods. For a given LHCII monomer trajectory we take a set of uncorrelated snapshots and for each calculate the population relaxation. The snapshots sample disorder in the inter-pigment excitonic couplings and the different minima in the original steered MD may reveal differences in the *average* couplings. We do not calculate the Chl excitation (site) energies *in situ* but simply take the average values reported in Müh et al. (2010). The reason for this is partly to spare computational expense and partly because these fluctuations have almost no effect on quenching (Balevicius and Duffy, 2020). To check the validity of the model we calculate the linear absorption (LA) and fluorescence (FL) profiles, examples of which are shown in Figures 1A,B, adding Gaussian disorder to the site energies to reproduce the broadening.

### 2.2. Relaxation Kinetics of Lutein in Pyridine

For Lut we adopt the Vibrational Energy Relaxation Approach (VERA) (Balevičius et al., 2018; Balevicius et al., 2019) to reproduce several independent spectral measurements. The details are discussed in Methods (and section C of the Supporting Information) but essentially the four singlet electronic states (|*S*_0_〉, |*S*_1_〉, |*S*_2_〉, |*S*_*n*_〉) are replaced by sets of vibronic states, ($|i_{a_{1}a_{2}}\rangle =|i\rangle |a_{1}\rangle^{i}|a_{2}\rangle^{i}$), where *i* is the electronic index and *a*_1_ and *a*_2_ are the vibrational quantum numbers associated with the high-frequency, optically-coupled *C* − *C* and *C* = *C* modes, respectively (Balevičius et al., 2018). For example, the state |0_00_〉 corresponds to the absolute ground state and |1_10_〉 to the first excited vibrational state of the C-C stretching mode on the first excited electronic state |*S*_1_〉. The LA is given by the sum of all vibronic transitions belonging to |*S*_0_〉 → |*S*_2_〉 (weighted by the Franck-Condon overlaps and the initial populations on |0_*a*_1_,*a*_2__〉) and is shown in Figure 1C alongside the experimental profile for Lut in pyridine. The fit is very good up to the blue edge of the first vibronic peak after which there is a deviation due to contributions from different geometrical conformers that are not accounted for in our model (Lukeš et al., 2011). The rise above 27,500cm^−1^ is a solvent artifact.

**Figure 2:**
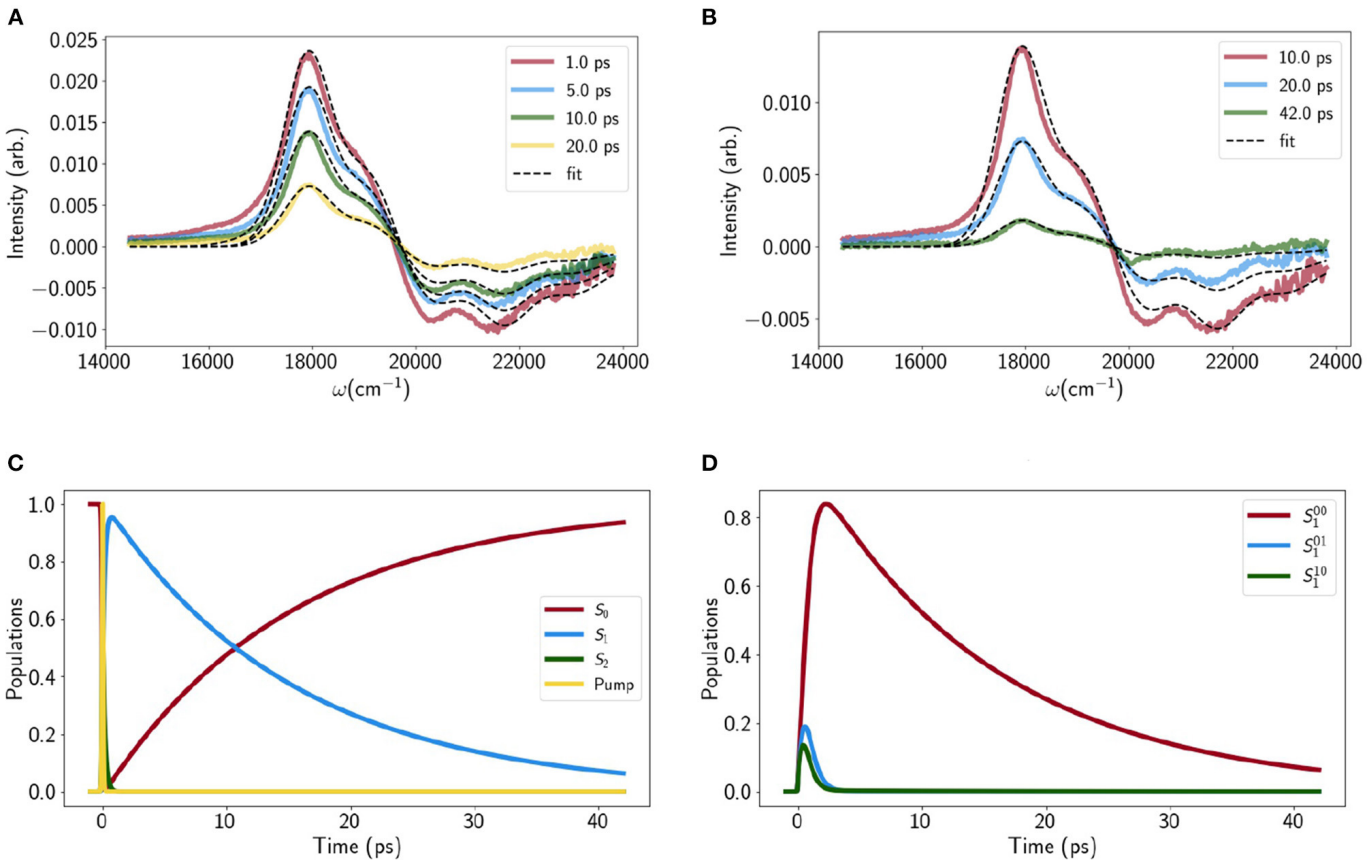
Transient absorption traces for **(A)** medium times and **(B)** long times, with experimental data shown in solid, colored lines and model fits shown as dashed lines. **(C)** The evolution of the total population on each electronic state as a function of time, along with the temporal shape of the pump pulse. Note that a large part of the *S*_2_ → *S*_1_ decay (green line) overlaps with the pump. **(D)** Population evolution of the 3 lowest vibrational levels on *S*_1_.

The static properties of *S*_1_ and all dynamical properties were obtained by fitting the VERA model to the TA of Lut in pyridine. Figures 2A,B show the calculated and experimental difference spectra at intermediate (1 − 20ps) and long (10 − 42ps) delay times, respectively. The sub-picosecond kinetics are not shown as they are less relevant to the final quenching model. The *S*_1_ Excited State Absorption (ESA, positive feature around 18,000cm^−1^) is well fit but there is some discrepancy for the Ground State Bleach (GSB, negative feature) at earlier times. While the fit can be improved by adjusting the *S*_2_ parameters, this disrupts the original LA fit. This could be linked to GSB-distorting local heating effects which have previously been reported (Balevicius et al., 2019) or simply an artifact. Either way it is the *S*_1_ parameters and kinetics that we are primarily interested in. All fitting parameters are reported in section A of the Supporting Information but there are a few key quantities:

- **The phononless** ***S***_**1**_ **energy**, $\varepsilon_{S_{1}}=14,050\;{\text{cm}}^{-1}$: We assumed the previously reported value during the fit to reduce the number of free parameters. Varying ε_*S*_1__ naturally ruins the fit but it can be recovered by adjusting other parameters (mainly ε_*S*_*n*__ and the dimensionless displacements between *S*_1_ and *S*_0_. As an independent check we calculated the *S*_0_ → *S*_1_ lineshape and compared it to the TPE of Lut in octanol (Walla et al., 2002). This is shown in Figure 1D and apart from a 150 cm^−1^ blue shift to account for the different solvent there is a reasonable visual agreement. However, we must note that the fit (nor the data, really) does not match the mirror image of the *S*_1_ FL line-shapes observed for Carts such as neurosporene and spheroidene Fujii et al. (1998). These have a much less defined vibronic structure and deconvolution suggests that the largest peak is the 0 − 2 line (|0_00_〉 → |1_01_〉 in our model) rather than 0 − 1. We found it impossible to reproduce such a lineshape while retaining any kind of fit to the TA and TPE data. This may be a limit of the displaced oscillator model but it was later suggested that *S*_1_ FL measurements may be distorted by the presence of cis-isomers (Christensen et al., 2007).
- **The** ***S***_**1**_ **lifetime**, 〈τ_*S*_1_ → *S*_0__〉 ≈ 14ps: Figure 2C shows the simulated evolution of total population on *S*_2_, *S*_1_ and *S*_0_. *S*_2_ → *S*_1_ internal conversion (IC) occurs on the ≈ 100fs timescale while *S*_1_ undergoes near mono-exponential decay in within the 10 − 20 ps range usually quoted for xanthophylls.
- **Vibrational relaxation on** ***S***_**1**_, 〈τ_vib−*S*_1__〉 ≈ 1ps: Figure 2D shows the simulated population evolution of the *S*_1_ vibronic levels |1_00_〉, |1_10_〉 and |1_01_〉. While it is difficult to assign a single lifetime to a multi-component process it is clear that vibrational relaxation on *S*_1_ is an order of magnitude faster than *S*_1_ IC but far from instantaneous.
- **Vibrational relaxation on** ***S***_**0**_, 〈τ_vib−*S*_0__〉 < 14ps: There is a very small transient population on |0_01_〉 and |0_10_〉 which reaches a peak at ≈ 9 ps (not shown) and makes a very small contribution to the blue shoulder on the *S*_1_ ESA. However, unlike Carts such as canthaxanthin and rhodoxanthin (Balevicius et al., 2019), the *S*_1_ IC is too slow to generate a vibrational population inversion on *S*_0_ and hence there is no S^*^-type signal (Balevicius et al., 2019).

### 2.3. Excitonic States and Intermolecular Couplings

**Figure 3:**
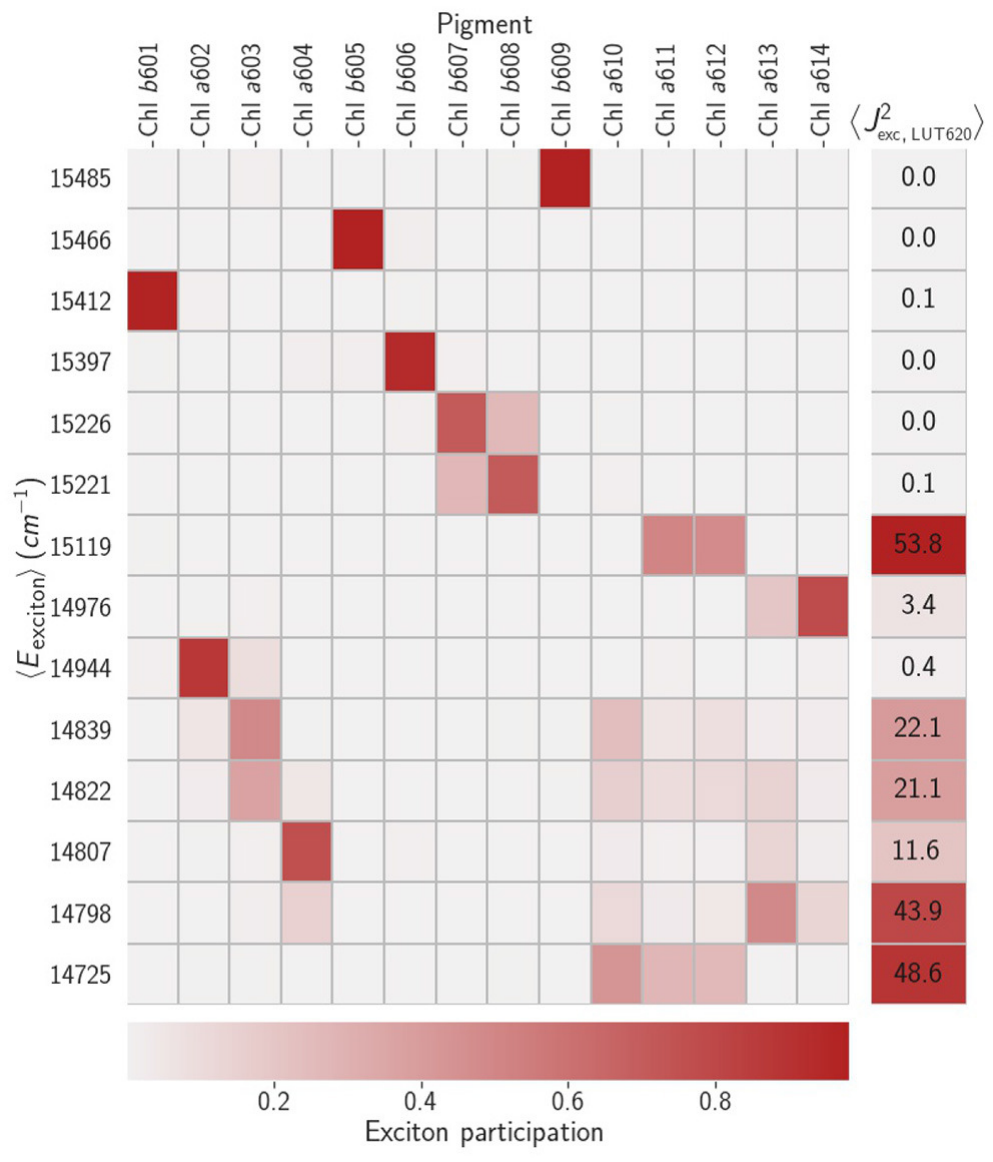
The grid shows the average energies, 〈*E*_*i*_〉, of the excitonic states and the average exciton participations, $\langle |c_{i}^{n}\left(t_{k}\right){|}^{2}\rangle$, for a typical minimum. The right-most column lists typical average values of the square couplings between the exciton states and the 0 − 0 transition on Lut. The coupling to higher vibronic transitions are simply weighted by the relevant Franck-Condon overlaps.

As a baseline we first simulated relaxation in the LHCII crystal structure (Liu et al., 2004) following protonation and minimization. The Chl-Chl couplings are essentially as previously reported (Novoderezhkin et al., 2004; Müh et al., 2010) and diagonalization leads to a set of exciton states that have already been described elsewhere (Novoderezhkin et al., 2004). Briefly, in the range 15,200 to 15,500cm^−1^ we find a set of almost single-molecule (unmixed) Chl *b* states. Between 14,700 and 15,200cm^−1^ are a set of excitonic states typically localized on dimers or trimers of Chl *a*. Particularly relevant to NPQ is the terminal emitter state at around 14,730 cm^−1^ which is localized on the Chl *a*610-*a*611-*a*612 domain closely associated with Lut1, which we label |TE^−^〉. There is also an analogous 'anti-bonding' state, |TE^+^〉, at around 15,120cm^−1^. This is shown diagrammatically in Figure 3.

**Figure 4:**
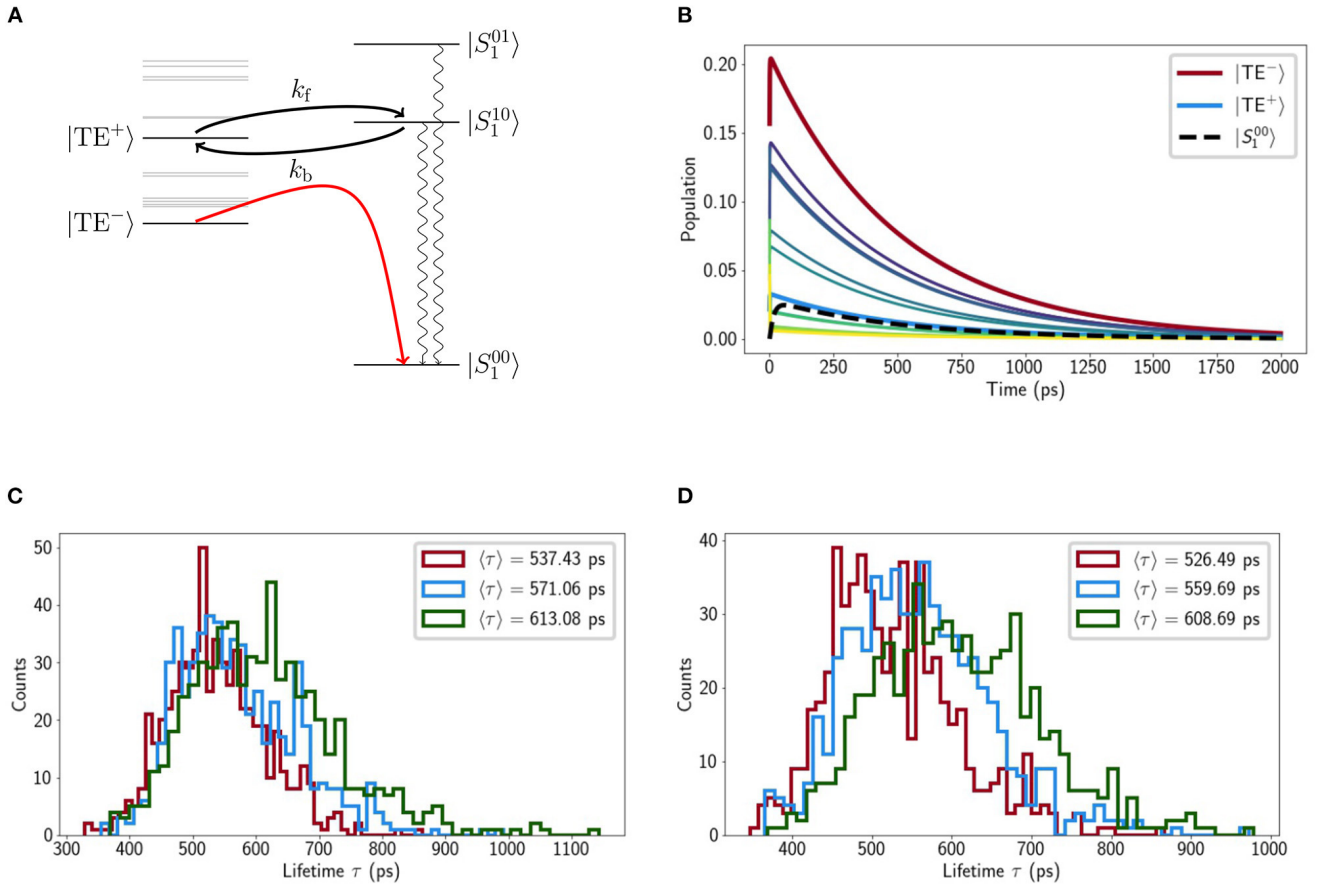
**(A)** Schematic representation of our model with the Chl excitonic manifold shown on the left and relevant carotenoid vibronic states on the right (energies are to scale). **(B)** Chl exciton populations calculated from the crystal structure as a function of time. The exciton states associated with the terminal emitter along with the population on lowest vibrational level of *S*_1_ ($|S_{1}^{00}\rangle$). Selected histograms of the mean excitation time 〈τ〉 of different LHCII minima (from the steered MD) for low **(C)** and neutral pH **(D)**, respectively.

We then consider the purely electronic Chl-Lut1 couplings, $J_{n,Lut}^{0}$ using Lut transition charges from Khokhlov and Belov (2019). In the site basis we have the same picture as previously reported (Chmeliov et al., 2015), weak (10 − 20cm^−1^) couplings to the terminal emitter Chls and negligible couplings otherwise. Excitonic mixing among the Chls naturally mixes these couplings, the strongest ($|J_{i,Lut}^{0}|\approx 7{\text{cm}}^{-1}$) being between Lut1 and |TE^−^〉 and |TE^+^〉. These small couplings justify (Balevicius and Duffy, 2020) our mixed kinetic model in which the Chl excitonic and Lut1 vibronic subsystems can exchange energy incoherently. When we model the relaxation kinetics the presence of Lut1 results in a decreased excitation lifetime of τ_ex_ ≈ 500 ps, compared to the unquenched value of 4ns (Pascal et al., 2005). The pathway is two-fold, involving fairly-reversible transfer from |TE^+^〉 to the near-resonant $|S_{1}^{10}\rangle =|1_{10}\rangle$ level and steep down-hill transfer from |TE^−^〉 to $|S_{1}^{00}\rangle =|1_{00}\rangle$ (see Figure 4A). Recent femtosecond stimulated Raman spectroscopy (Artes Vivancos et al., 2020) has also suggested that vibrationally excited states on Lut 1 can participate in light-harvesting by internal conversion from S2 and then energy transfer to the chlorophylls, which agrees well with our observation that the |1_10_〉 state is resonant with |TE+〉. The transfer is typically slow, however. For example, the rate constant for transfer from |TE^−^〉 to $|S_{1}^{00}\rangle$ is $k_{S_{1}^{00},{\text{TE}}^{-}}^{-1}\approx 300$ ps. There are, however, several pathways that contribute, involving other exciton states and the $|S_{1}^{10}\rangle$ vibronic level, resulting is a net timescale of ≈ 100 ps. This is too slow for any transient accumulation of population on $|S_{1}^{00}\rangle$ (see Figure 4B).

While these results are essentially identical to those previously reported (Chmeliov et al., 2015), it is important to realize that the absolute value of τ_ex_ may not be quantitatively accurate, due to the fact that the Chl-Lut couplings are derived from unscaled *S*_1_ transition charges from quantum chemical calculations (Khokhlov and Belov, 2019), but the relative changes in lifetime between minima are meaningful.

### 2.4. Exploring the LHCII Potential Energy Surface

We calculated the average, *relative* mean excitation times for several minima identified by a previous steered MD study (Daskalakis et al., 2020). It was reported that different monomers within the same LHCII trimer could access different conformational states and so we consider the monomer in our calculations. The minima are broadly classified into ‘low pH’ and ‘neutral pH’ depending on the protonation state of several lumen-exposed residues. In all minima there were fluctuations in the *snapshot lifetimes*, 300 < τ_*ex*_ < 1000ps, but the average value varies little within the range 500 < 〈τ_*ex*_〉 < 600ps (see Figures 4C,D).

It is premature to say that ‘all of these minima are quenched’ but we can state that there is no evidence of a simple, purely-geometric switch between states with significantly different lifetimes. We find (as previously noted Fox et al., 2017) that τ_ex_ is correlated with Lut1-Chl *a*612 coupling but the coupling is not sufficiently sensitive to the small movements of the pigments to produce any kind of functional transition.

### 2.5. *S*_1_ Excitation Energy and Asymmetry Between Lut1 and Lut2

**Figure 5:**
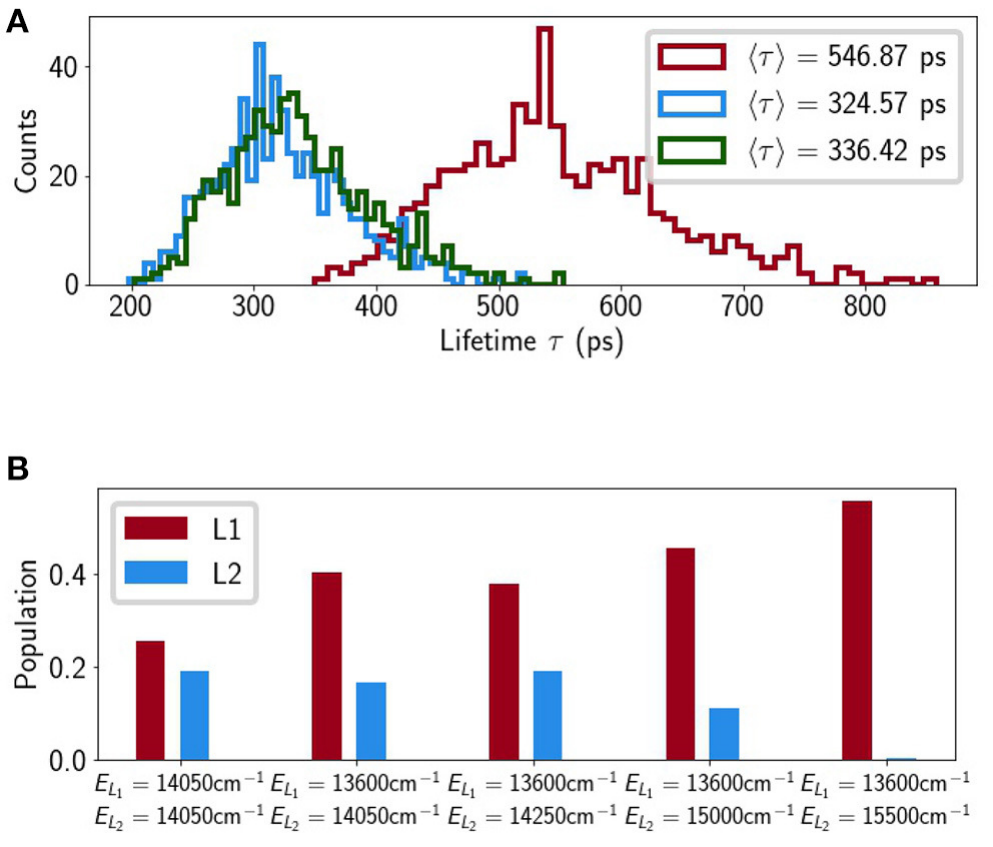
**(A)** Lifetime histograms with *E*_*S*_1__ set to 14,050 cm^−1^ (red), 13,600 cm^−1^ (blue) and 14,750 cm^−1^ (green), and **(B)** average populations on L1 and L2 at τ, where *E*_*L*_*i*__ denotes the $S_{1}^{00}$ energy assigned to Lut *i*.

If we trust the TA-derived value of $\varepsilon_{S_{1}}=14,050{\text{cm}}^{-1}$ then the relative arrangement of excitonic and vibronic levels is notable (see Figure 4A). |*TE*^+^〉 and $|S_{1}^{10}\rangle$ are near-resonant but since |*TE*^+^〉 acquires little exciton population at room temperature (see Figure 4B) this is not a very effective pathway for quenching. The terminal emitter state, |*TE*^−^〉, lies almost precisely in the middle of $|S_{1}^{00}\rangle$ and $|S_{1}^{10}\rangle$ meaning any reasonable shift in the relative energy actually *increases* the quenching. This is shown in Figure 5A where we alter ε_*S*_1__ to bring either $|S_{1}^{00}\rangle$ ($\varepsilon_{S_{1}}=14,750{\text{cm}}^{-1}$) or $|S_{1}^{10}\rangle$ ($\varepsilon_{S_{1}}=13,600{\text{cm}}^{-1}$) into resonance with |*TE*^−^〉. In both cases 〈τ_ex_〉 drops by around 50% to roughly 300 ps. Within the smaller range we find that changes in the energy of $\varepsilon_{S_{1}}=14,050\pm 300{\text{cm}}^{-1}$, i.e., within the error bar of the reported value, the largest decrease is by about 25%. For $\varepsilon_{S_{1}}>18,000{\text{cm}}^{-1}$ the quenching disappears completely (〈τ_ex_〉 → Γ_Chl_ = 4 ns), as has been previously reported (Lapillo et al., 2020). This is simply because there is no energetic overlap between the two sub-systems and energy transfer between them is impossible by construction.

Although we initially excluded Lut2 we then put it back into the model, assuming the same transition charges and, initially, the same excitation energy as Lut1. The binding pocket of Lut2 (superficially) mirrors that of Lut1 with weak couplings to Chls *a*603 and *a*604 which participate in several excitonic states between |*TE*^−^〉 and |*TE*^+^〉. This leads to a 40% decrease in lifetime relative to the Lut1-only model. Figure 5B shows the excitation population on the Lut ground state at time *t* = τ_ex_ and we see that when $\varepsilon_{S_{1}}^{\text{Lut2}}=\varepsilon_{S_{1}}^{\text{Lut1}}=14,050{\text{cm}}^{-1}$ Lut2 is almost as effective a quencher as Lut1. This is contrary to the observed features of NPQ and the known properties of Lut2. The initial TA measurements that lead to the proposal of a Lut-mediated NPQ identified Lut1 as the sole quencher (Ruban et al., 2007). This is likely because Lut2 has a significantly distorted electronic structure relative to that in solution. The *S*_2_ excitation energy of Lut 2, $\varepsilon_{S_{2}}^{\text{Lut2}}$, is significantly lower than $\varepsilon_{S_{2}}^{\text{Lut1}}$ (Son et al., 2020) and if this shift is caused by twisting of the backbone then it is likely accompanied by a concomitant upward-shift in $\varepsilon_{S_{1}}^{\text{Lut1}}$ (Wei et al., 2019; Artes Vivancos et al., 2020). In fact, recent ultra-broadband 2D measurements on LHCII identified a dark state (termed *S*_*X*_), lying above the Chl a *Q*_*y*_ band, which belongs exclusively to Lut2 (Son et al., 2019). This is most likely a strongly-distorted *S*_1_. Figure 5B shows that quenching by Lut2 can be completely abolished if we introduce some energetic asymmetry between Lut1 and Lut2. The shifts are not actually that large with $\varepsilon_{S_{1}}^{\text{Lut1}}=13,600{\text{cm}}^{-1}$ being almost within the error bars of the measured value of 14,050 ± 300cm^−1^ and $\varepsilon_{S_{1}}^{\text{Lut2}}=15,000-15,500{\text{cm}}^{-1}$ being roughly in the region of the proposed *S*_*X*_ state. It is important to note that this is not a rigorous analysis, which would require independent, *in situ* parameterization of Lut1 and Lut2. However, it points to an energetic sensitivity in the quenching pathway(s) that was absent in previous models.

## 3. Discussion

The essence of the quenching mechanism investigated here (and previously proposed Balevičius et al., 2017) is that trivial geometric modulation of Chl-Lut coupling is sufficient to drive the system between quenched and unquenched states. This appears to be incorrect, as the complex simply does not seem to possess the conformational flexibility to induce significant changes in the coupling. We are not saying that the different minima do not represent different functional states or that carotenoids are not involved in quenching, merely that our model does not capture its key features. There are several possible **quenching scenarios** that can be discussed.

### 3.1. NPQ May Involve Modulation of the Properties of *S*_1_

The point of this study was to try and cast this problem in terms of experimental parameters, specifically the *S*_1_ energy, vibronic structure and relaxation dynamics. While the TA fits seem reliable, the data is for Lut in pyridine and obviously there is a question of whether this can be applied to Lut in protein. Actually, the default value of $\varepsilon_{S_{1}}=14,050{\text{cm}}^{-1}$ was taken from NIR measurements on Lut in LHCII, although the error bars are quite big (±300cm^−1^) and the study did not compare quenched and unquenched configurations (Polívka et al., 2002). An earlier NPQ model proposed that quenching was induced by bringing the Chl *Q*_*y*_ band and *S*_1_ into resonance (Holleboom and Walla, 2014), which would require either $\varepsilon_{S_{1}}^{00}$ or $\varepsilon_{S_{1}}^{10}\approx 15,000{\text{cm}}^{-1}$. Balevičius Jr. and Duffy recently provided a very general physical argument as to why fine tuning of this energy gap cannot modulate quenching (Balevicius and Duffy, 2020) and showed that significant quenching is possible for large energy gaps, even if the quenching state lies above the donor state. *S*_1_ has to be quite far above the *Q*_*y*_ band to abolish quenching, as was recently proposed by Lapillo et al. (2020). They reported a sharp dependence of the EET rate (and overall quenching) on the energy gap when $\varepsilon_{S_{1}}\approx 18,000{\text{cm}}^{-1}$. This is simply because at this point the *Q*_*y*_ band coincides with the steep red edge of the *S*_1_ lineshape. However, as they point out, ε_*S*_1__ is not a free parameter and a protein-induced blue-shift of 14,050 → 18,000cm^−1^ (712 → 555 nm) would be quite large. Saccon et al. recently performed TA measurements on quenched LHCII immobilized in polyacrylamide gel (a model for NPQ) (Saccon et al., 2020) and found that linear excitation of Lut (i.e., via *S*_2_) produces the usual *S*_1_ → *S*_*n*_ ESA at $\varepsilon_{S_{n}}-\varepsilon_{S_{1}}\approx 18,500{\text{cm}}^{-1}$ (540 nm). This is reasonably close to the value in pyridine, $\varepsilon_{S_{n}}-\varepsilon_{S_{1}}\approx 17,900{\text{cm}}^{-1}$ (558 nm). Of course this is an indirect measurement and a massive shift in ε_*S*_1__ could be hidden by a correlated shift in ε_*S*_*n*__. However, this would have a significant affect on the ESA formation and decay kinetics which is not observed.

### 3.2. NPQ May Involve Non-coulomb Interactions and/or Non-adiabatic Inter-molecular States

When estimating Chl-Chl couplings the *Q*_*y*_ transition density/charges are typically re-scaled to reproduce an experimentally determined oscillator strength (Knox and Spring, 2003; Müh et al., 2010). This is clearly not an option for *S*_1_ (for which a non-zero oscillator strength has never been measured) and therefore the absolute values of *S*_1_ transition charges and the Chl-Lut couplings are difficult to estimate. For the planar geometry of Lut the published transition atomic charges (Khokhlov and Belov, 2019) yield a dipole moment of |μ_*S*_1__| ≈ 0.1 − 0.2*D* which, although very small, is nonzero (|μ_*Q*_*y*__| ≈ 3 − 5*D* for comparison Knox and Spring, 2003). It is possible that the amplitude of the *S*_1_ transition density is over-estimated and therefore so are the Coulomb couplings, *Q*_*y*_ → *S*_1_ transfer rates, and overall level of quenching. In fact, given that there appears to be insufficient conformational flexibility in LHCII to switch this Coulomb-mediated quenching off, it may be an artifact. The reason that it was initially considered promising was that it qualitatively matched the NPQ scheme proposed by Ruban et al. in 2007, based on TA measurements of quenched LHCII aggregates (Ruban et al., 2007). The role of *S*_1_ was implied by global target analysis of the kinetics rather than any visually detectable *S*_1_ signal and so must be treated with caution. *Direct* observation of *S*_1_-mediated quenching was later reported for the cyanobacterial *High light inducible proteins* (Hlips) which are ancestors of LHCII (Staleva et al., 2015). Hlips are perpetually quenched by ≈ 2 ps (hence observable) EET from a small pool of Chl a to β-carotene in one of the central binding pockets that are analogous to L1 and L2 in LHCII. More recently, sub-picosecond EET to Lut1 was directly observed in LHCII via ultra-fast 2D spectroscopy (Son et al., 2020). In both the EET is much faster than predicted by this or any of the previous models and it is difficult to see how such fast transfer could be Coulomb-mediated and be in any way switchable or involve an optically-forbidden transition. Cignoni et al. provide a possible answer via a detailed QM/MM study of CP29 in which short-range interactions (exchange, overlap, etc.) were found to make large contributions to the Chl-Cart couplings (Cignoni et al., 2021). These are naturally far more sensitive to minor conformational changes than the long-range Coulomb interactions.

The picture gets even more complicated when one considers quenched LHCII in gel. It was recently shown that excitation of *Q*_*y*_ results in the immediate appearance of a large-amplitude positive peak at 19,417cm^−1^ (515 nm) which we'll label *A*_515_ (Saccon et al., 2020). This is not merely a shifted *S*_1_ as direct excitation of Lut gave the usual *S*_1_ → *S*_*n*_ ESA at 18,500cm^−1^ (540 nm), although *A*_515_ is detectable at later times and may simply be initially hidden by *S*_1_. This suggests that *S*_2_ → *S*_1_ and *S*_2_ → *A*_515_ are competing pathways. *A*_515_ is in the region of the S^*^ signal which some people suggest is either a distorted *S*_1_ or a dipole-forbidden singlet electronic state lying below *S*_1_ (Mascoli et al., 2019). That argument aside, since the Chl ESA is typically flat and featureless, it seems reasonable to assume that *A*_515_ is associated with the Cars, although it is difficult to assign it solely to Lut1. *A*_515_ is independent of whether it is Chl *a* or Chl *b* that is excited and the GSB bands in the *S*_2_ region (< 500 nm) looks very different to the classic *S*_2_ GSB. This all suggests some type of delocalized quenching pathway that involves several Carts and possibly even some non-adiabatic intermolecular states not accessible simply by exciting *S*_2_. This is exactly the picture emerging from the elaborate QM/MM models of CP29 being reported by Mennucci et al. (Cupellini et al., 2020; Lapillo et al., 2020).

### 3.3. Quenching Requires Hydrophobic Mismatch and Aggregation

It is possible that the conformational switch cannot be revealed by simulating a single LHCII monomer/trimer in a lipid bilayer. *In vitro* quenching is induced by low detergent concentration which in solution leads to aggregation. LHCII aggregates are the original model system for studying NPQ (Horton et al., 1991) and there is compelling evidence that some form of aggregation or clustering in the membrane is part of the *in vivo* mechanism (Johnson et al., 2011). Key to this is PsbS, with over-expression observed to enhance LHCII clustering and its absence frustrating it (Goral et al., 2012). Recent simulations show that PsbS's lumen-exposed side is covered in titratable residues with protonation causing an unfolding of a specific region implicated in protein-protein interactions (Liguori et al., 2019). Other studies have shown that it is capable of interacting with the minor PSII antenna complexes (Daskalakis, 2018), possibly helping LHCII to partially detach from the reaction center complex and form the quenching clusters. It has also been suggested that active PsbS has an affinity for certain lipids, altering local membrane composition and causing the hydrophobic mismatch that drives aggregation/clustering (Daskalakis et al., 2019; Ruban and Wilson, 2020).

The role of external interactions on LHCII conformation was (at least partially) considered in the metadynamical simulations used in this work (Daskalakis et al., 2020). They were performed by first calculating the free energy surface (FES) of LHCII while considering a wide range of external stimuli, such as ΔpH and interactions with PsbS. The next step involved steering MD simulations of an LHCII trimer around this FES in order to reach the minima. Equilibrium MD trajectories were then performed on an isolated trimer once each minimum was reached. Due to the absence of external stimuli in these equilibrium trajectories, it is possible that the configuration space even at these minima still favors the quenched conformation over the unquenched.

## 4. Methods

### 4.1. TA Measurements of Lutein in Pyridine

All transient absorption data were measured with a spectrometer described in detail in Saccon et al. (2020). Lutein (Sigma Aldrich) was dissolved in spectroscopic grade pyridine to yield an optical density of ≈ 0.2 mm^−1^ at the absorption maximum. The sample was placed in a 2 mm path-length quartz cuvette equipped with a micro-stirrer to avoid sample degradation during measurement. The mutual polarization of the excitation and probe beams was set to the magic angle (54.7°) and excitation intensity was kept below 1014 photons pulse^−1^cm^−2^.

### 4.2. The Chlorophyll Exciton Manifold

Modeling of energy relaxation within the chlorophylls is carried out according to previous work (Novoderezhkin et al., 2004; Malý et al., 2016) and is described in detail in section B of the Supporting Information. Briefly, for a single uncorrelated MD snapshot (at time *t*_*k*_) the relevant system of Chl excited (*Q*_*y*_) states is determined by the usual spin-boson Hamiltonian,

(1) $$\begin{array}{r} H\left(t_{k}\right)=\underset{n}{\sum }E_{n}|n\rangle \langle n|+\underset{m\neq n}{\sum }J_{mn}\left(t_{k}\right)|m\rangle \langle n| \end{array}$$

where {|*m*〉} is the 'site' basis of uncoupled single-molecule excitations, {*E*_*m*_} are the site (excitation) energies and {*J*_*mn*_(*t*_*k*_)} are the resonance couplings. {*J*_*mn*_(*t*_*k*_)} are calculated as the sum of pairwise Coulomb interactions between transition atomic charges, {*q*_α_},

(2) Jmn(tk)=14πε∑α∈mβ∈nqαqβ|rα(tk)-rβ(tk)|

where ε = ε_*r*_ε_0_ = 2ε_0_. Both {*E*_*n*_} and *q*_α_ are taken from Müh et al. (Madjet et al., 2006; Renger et al., 2007). Equation (1) is then diagonalised to give the exciton basis,

(3) $$\begin{array}{r} |i\rangle =\underset{n}{\sum }c_{n}^{i}|n\rangle \end{array}$$

where $c_{n}^{i}$ are the participation coefficients of each pigment state, |*n*〉 for a given exicton state, |*i*〉. Site energies, oscillator strengths and couplings to Cart vibronic levels are also mixed. The exciton states are initially populated according to their oscillator strengths and relaxation is modeled using the approach outlined in Novoderezhkin et al. (2004). The population relaxation rates are given by,

(4) $$\begin{array}{r} k_{ij}=\underset{n}{\sum }{\left|c_{n}^{i}\right|}^{2}{\left|c_{n}^{j}\right|}^{2}\left(1+\coth\left(\frac{\hbar \omega_{ij}}{2k_{B}T}\right)\right)C_{n}^{\prime \prime }\left(\omega_{ij}\right) \end{array}$$

where $C_{n}^{\prime \prime }\left(\omega \right)$ is the spectral density of bath-induced site energy fluctuations and ω_*ij*_ is the gap between the zero-phonon lines of excitons *i* and *j*. The ansatz spectral density (Novoderezhkin et al., 2004) is assumed throughout. For a single uncorrelated snapshot along a trajectory (at time *t*_*k*_) the instantaneous LA and FL spectra are given by

(5) $$\begin{array}{r} A\left(\omega ;t_{k}\right)\propto \omega \underset{i}{\sum }\chi_{i}\left(\omega ;t_{k}\right) \end{array}$$

and,

(6) $$\begin{array}{r} F\left(\omega ;t_{k}\right)\propto \omega^{3}\underset{i}{\sum }{\tilde{\chi }}_{i}\left(\omega ;t_{k}\right)P_{i}\left(\infty ;t_{k}\right) \end{array}$$

where {χ_*i*_(ω, *t*_*k*_)} and $\left\{{\tilde{\chi }}_{i}\left(\omega ;t_{k}\right)\right\}$ are the instantaneous LA and FL line-shapes, respectively and {*P*_*i*_(∞; *t*_*k*_)} are the steady state populations of the exciton states. The true LA and FL for a particular minima are given by averaging over a trajectory, {*J*_*mn*_(*t*_*k*_)}, and then again over several instances of Gaussian disorder in the Chl site energies, {*E*_*m*_}.

### 4.3. The Carotenoid Vibronic Subsystem

The full VERA (Balevičius et al., 2018; Balevicius et al., 2019) Hamiltonian is,


(7a) $$\begin{array}{r} H_{\text{car}}=H_{\text{S}}+H_{\text{B}}+H_{\text{SB}}^{\text{IVR}}+H_{\text{SB}}^{\text{IC}} \end{array}$$

(7b) =∑ia1a2εia1a2|ia1,a2〉〈ia1,a2|

(7c) $$\begin{array}{r} +\underset{\kappa }{\sum }\left(\frac{p_{\kappa }^{2}}{2m_{\kappa }}+\frac{m_{\kappa }\omega_{\kappa }^{2}x_{\kappa }^{2}}{2}\right) \end{array}$$

(7d) $$\begin{array}{r} +[\underset{i,\kappa }{\sum }c_{i\kappa }x_{\kappa }\sqrt{a_{1}+1}(|i_{a_{1},a_{2}}\rangle \langle i_{a_{1}+1,a_{2}}|+|i_{a_{1}+1,a_{2}}\rangle \langle i_{a_{1},a_{2}}|)\\ \;+\underset{i,\kappa }{\sum }c_{i\kappa }x_{\kappa }\sqrt{a_{2}+1}(|i_{a_{1},a_{2}}\rangle \langle i_{a_{1},a_{2}+1}|+|i_{a_{1},a_{2}+1}\rangle \langle i_{a_{1},a_{2}}|)] \end{array}$$

(7e) +[∑i,κa1,a2b1,b2fiκxκ(∏α=1,2Fiaα,i+1bαα)|ia1a2〉〈i+1b1b2|    +∑i,κa1,a2b1,b2fiκxκ(∏α=1,2Fi+1aα,ibαα)|i+1a1a2〉〈ib1b2|]

Equation (7b) is the Hamiltonian of the system ($H_{\text{S}}$) of uncoupled vibronic levels, {|*i*_*a*_1_*a*_2__〉}, where


(8) $$\begin{array}{r} \varepsilon_{i}^{a_{1}a_{2}}=\epsilon_{i}+\epsilon_{a_{1},a_{2}}=\epsilon_{i}+\left(a_{1}+\frac{1}{2}\right)\hbar \omega_{1}+\left(a_{2}+\frac{1}{2}\right)\hbar \omega_{2} \end{array}$$

is the sum of the electronic, ϵ_*i*_ and vibrational energies. $H_{\text{B}}$ is the bath Hamiltonian which is composed of a large set of harmonic oscillators representing the non-optical modes of the Cart itself plus librations, solvent modes, etc. We split this into two parts, $H_{\text{SB}}^{\text{IVR}}$ and $H_{\text{SB}}^{\text{IC}}$. $H_{\text{SB}}^{\text{IVR}}$ describes the bath-induced couplings between adjacent vibrational levels of the optical modes and are therefore responsible for vibrational relaxation on the electronic states. {*c*_*iκ*_} are the coupling constants and {*x*_κ_ the bath mode displacements. Energy (mostly) relaxes into the non-optical modes of the Cart and therefore reflects Intramolecular Vibrational Redistribution (IVR). Note that there is no population transfer between the two optically-coupled modes. $H_{\text{SB}}^{\text{IC}}$ couples different electronic states and is therefore responsible for Internal Conversion (IC). It is characterized by coupling constants, *f*_*iκ*_, and the Franck-Condon (FC) overlaps,

(9) $$\begin{array}{r} F_{i_{a_{\alpha }},j_{b_{\alpha }}}^{\alpha }={\;}^{i}{\langle a_{\alpha }|b_{\alpha }\rangle }^{j} \end{array}$$

If we assume that the frequencies of the optical modes (ω_α_) are independent of the electronic state (i.e., no Duschinsky rotations) then $\left\{F_{i_{a_{\alpha }},j_{b_{\alpha }}}^{\alpha }\right\}$ are entirely determine by their relative dimensionless displacements, $\left\{d_{\alpha }^{ij}\right\}$. The relaxation dynamics are obtained by a second-order perturbative treatment of $H_{\text{SB}}^{\text{IVR}}$ and $H_{\text{SB}}^{\text{IC}}$. The resulting equations of motion are rather complicated and are listed in section C of the Supporting Information. The various IVR, $k_{\alpha }^{\pm }$, and IC, $k_{a_{1}a_{2},b_{1}b_{2}}^{ij}$, rate constants are defined in terms of Drude-type spectral density functions $C_{i_{\alpha }}^{\prime \prime }\left(\omega \right)$ and $C_{i_{\alpha },j_{\alpha }}^{\prime \prime }\left(\omega \right)$. We therefore have a large set of fitting parameters including electronic, {ϵ_*i*_} and vibrational, ϵ_*a*_1_*a*_2__, energies, modes frequencies, ω_α_, mode displacements, $d_{\alpha }^{ij}$, and the reorganization energies, λ_*i*_α__, λ_*i*_α_, *j*_α__, and dephasing frequencies, γ_*i*_α__, γ_*i*_α_, *j*_α__. Solving the dynamics yields a set of vibronic populations, $n_{a_{1}a_{2}}^{i}\left(t\right)$, which are used to calculation the TA difference spectrum as a combination of ESA, GSB and stimulated emission (SE) components,

(10) $$\begin{array}{r} \Delta A\left(\omega ,t\right)=A_{\text{ESA}}\left(\omega ,t\right)-A_{\text{SE}}\left(\omega ,t\right)-A_{\text{GSB}}\left(\omega ,t\right) \end{array}$$

which are given by,

(11) $$\begin{array}{r} A_{X}\left(\omega ,t\right)=\underset{i,a_{1},a_{2}}{\sum }n_{a_{1}a_{2}}^{i}\left(t\right)I_{a_{1}a_{2},X}\left(\omega \right) \end{array}$$

where *I*_*a*_1_*a*_2_, *X*_(ω) are FC-weighted Gaussian/Lorentzian lineshape functions that account for line-broadening.

### 4.4. Energy Transfer Between the Chlorophyll and Lutein Subsystems

Having parameterized the subsystems separately we can now couple them via the calculated resonance couplings, $J_{n,\text{Lut}}^{0}\left(t_{k}\right)$. We make two assumptions. Firstly, since the inter-pigment couplings between the Chls and Lut is an order of magnitude smaller than the nearest-neighbor chlorophyll couplings (there is essentially no coupling between Lut1 and Lut2), we treat the Chl-Lut system as two weakly-interacting subsystems and assume that energy transfer proceeds incoherently (Balevicius and Duffy, 2020). Secondly, since there is almost no accumulation of vibronic population on the ground state (‘hot’ ground state), we do not explicitly include the Chl or Lut ground states in the dynamics. *S*_1_ can decay to higher vibrational levels on *S*_0_ but excitation proceeds from $|S_{0}^{00}\rangle =|0_{00}\rangle$. Essentially, we are assuming instantaneous IVR on the ground state. The couplings in the exciton basis are given by,

(12) $$\begin{array}{r} J_{i,\text{Lut}}^{0}\left(t_{k}\right)=\overset{n_{\text{Chl}}}{\underset{n=1}{\sum }}c_{n}^{i}J_{n,\text{Lut}}^{0}\left(t_{k}\right) \end{array}$$

where $\left\{J_{n,\text{Lut}}^{0}\left(t_{k}\right)\right\}$ are the purely electronic Chl-Lut couplings. The rate of transfer from Chl exciton state |*i*〉 to Lut vibronic level $|S_{1}^{a_{1}a_{2}}\rangle =|1_{b_{1}b_{2}}\rangle$ is given by the Fermi Golden Rule,

(13) $$\begin{array}{r} k_{i\to \left(0,0,b_{1},b_{2}\right)}\left(t_{k}\right)=2\pi \left({\left|\underset{\alpha =1,2}{\prod }F_{0_{0_{\alpha }},1_{b_{\alpha }}}^{\alpha }\right|}^{2}\right){\left|J_{i,\text{Lut}}^{0}\left(t_{k}\right)\right|}^{2}\\ \;\times \int_{-\infty }^{\infty }d\omega {\tilde{\chi }}_{i}^{\prime }\left(\omega ;\omega_{i0}-\lambda_{i}\right)\sigma \left(\omega ;\Delta_{b_{\alpha }0_{\alpha }}^{10},\Delta \omega_{10}\right) \end{array}$$

where $\Delta_{b_{\alpha }a_{\alpha }}^{ij}=\left(\epsilon_{i}+\epsilon_{b_{1},b_{2}}\right)-\left(\epsilon_{j}+\epsilon_{a_{1},a_{2}}\right)$ is the vibronic energy gap, ${\tilde{\chi }}_{i}^{\prime }$ is the normalized excitonic fluorescence lineshape and $\sigma \left(\omega ;\Delta_{b_{\alpha }0_{\alpha }}^{10},\Delta \omega_{10}\right)$ is the normalized vibronic Gaussian lineshape of width $\Delta \omega_{10}=1070{\text{cm}}^{-1}$ determined by the TA fit. The backward rate is similarly defined and Boltzmann factors are added to uphill rates to enforce the detailed balance condition.

## Data Availability Statement

The original contributions presented in the study are included in the article/Supplementary Material, further inquiries can be directed to the corresponding author.

## Author Contributions

CD, CG, and TW devised the project. TP provided TA data and assisted with data fitting. CD performed TA data fitting. VD provided steered MD data. TW contributed pilot MD data for development of model. All calculations and overall model development by CG. CG and CD wrote the draft, all authors took part in editing.

## Conflict of Interest

The authors declare that the research was conducted in the absence of any commercial or financial relationships that could be construed as a potential conflict of interest.

## Publisher's Note

All claims expressed in this article are solely those of the authors and do not necessarily represent those of their affiliated organizations, or those of the publisher, the editors and the reviewers. Any product that may be evaluated in this article, or claim that may be made by its manufacturer, is not guaranteed or endorsed by the publisher.

## References

[B1] Ahn T. K. Avenson T. J. Ballottari M. Cheng Y.-C. Niyogi K. K. Bassi R.. (2008). Architecture of a charge-transfer state regulating light harvesting in a plant antenna protein. Science 320, 794–797. 10.1126/science.1154800 18467588

[B2] Andreussi O. Knecht S. Marian C. M. Kongsted J. Mennucci B. (1993). Carotenoids and light-harvesting: From dft/mrci to the tamm-dancoff approximation. J. Chem. Theory Comput. 11, 655–666. 10.1021/ct5011246 26579601

[B3] Aro E. M. Virgin I. Andersson B. (1993). Photoinhibition of photosystem ii. inactivation, protein damage and turnover. Biochim. Biophys. Acta 1143, 113–134. 10.1016/0005-2728(93)90134-2 8318516

[B4] Artes Vivancos J. M. van Stokkum I. H. M. Saccon F. Hontani Y. Kloz M. Ruban A.. (2020). Unraveling the excited-state dynamics and light-harvesting functions of xanthophylls in light-harvesting complex ii using femtosecond stimulated raman spectroscopy. J. Am. Chem. Soc. 142, 17346–17355. 10.1021/jacs.0c04619 32878439 PMC7564077

[B5] Balevičius V. Jr. Fox K. F. Bricker W. P. Jurinovich S. Prandi I. G.. (2017). Fine control of chlorophyll-carotenoid interactions defines the functionality of light-harvesting proteins in plants. Sci. Rep. 7, 13956. 10.1038/s41598-017-13720-6 29066753 PMC5655323

[B6] Balevičius V. Jr. Lincoln C. N. Viola D. Cerullo G. Hauer J.. (2018). Effects of tunable excitation in carotenoids explained by the vibrational energy relaxation approach. Photosynth. Res. 135, 55–64. 10.1007/s11120-017-0423-6 28741055

[B7] Balevicius V. Duffy C. D. P. (2020). Excitation quenching in chlorophyll-carotenoid antenna systems: ‘coherent’ or ‘incoherent’. Photosynth. Res. 144, 301–315. 10.1007/s11120-020-00737-8 32266612 PMC7239839

[B8] Balevicius V. Wei T. Tommaso D. D. Abramavicius D. Hauer J. Polívka T.. (2019). The full dynamics of energy relaxation in large organic molecules: from photo-excitation to solvent heating. Chem. Sci. 10, 4792–4804. 10.1039/C9SC00410F 31183032 PMC6521204

[B9] Belgio E. Duffy C. D. P. Ruban A. V. (2013). Switching light harvesting complex ii into photoprotective state involves the lumen-facing apoprotein loop. Phys. Chem. Chem. Phys. 15, 12253–12261. 10.1039/c3cp51925b 23771239

[B10] Bode S. Quentmeier C. C. Liao P.-N. Hafi N. Barros T. Wilk L.. (2009). On the regulation of photosynthesis by excitonic interactions between carotenoids and chlorophylls. Proc. Natl. Acad. Sci. U.S.A. 106, 12311–12316. 10.1073/pnas.0903536106 19617542 PMC2714278

[B11] Chmeliov J. Bricker W. P. Lo C. Jouin E. Valkunas L. Ruban A. V.. (2015). An ‘all pigment’model of excitation quenching in lhcii. Phys. Chem. Chem. Phys. 17, 15857–15867. 10.1039/C5CP01905B 26017055

[B12] Christensen R. L. Galinato M. G. I. Chu E. F. Fujii R. Hashimoto H. Frank H. A. (2007). Symmetry control of radiative decay in linear polyenes: low barriers for isomerization in the s1 state of hexadecaheptaene. J. Am. Chem. Soc. 129, 1769–1775. 10.1021/ja0609607 17284007 PMC2518222

[B13] Cignoni E. Lapillo M. Cupellini L. Acosta Gutierrez S. Gervasio F. L. Mennucci B. (2021). A different perspective for nonphotochemical quenching in plant antenna complexes. Nat. Commun. 12, 7152. 10.1038/s41467-021-27526-8 34887401 PMC8660843

[B14] Cupellini L. Calvani D. Jacquemin D. Mennucci B. (2020). Charge transfer from the carotenoid can quench chlorophyll excitation in antenna complexes of plants. Nat. Commun. 11, 662. 10.1038/s41467-020-14488-6 32005811 PMC6994720

[B15] Daskalakis V. (2018). Protein-protein interactions within photosystem ii under photoprotection: the synergy between cp29 minor antenna, subunit s (psbs) and zeaxanthin at all-atom resolution. Phys. Chem. Chem. Phys. 20, 11843–11855. 10.1039/C8CP01226A 29658553

[B16] Daskalakis V. Papadatos S. Kleinekathöfer U. (2019). Fine tuning of the photosystem ii major antenna mobility within the thylakoid membrane of higher plants. Biochim. Biophys. Acta 1861, 183059. 10.1016/j.bbamem.2019.183059 31518553

[B17] Daskalakis V. Papadatos S. Stergiannakos T. (2020). The conformational phase space of the photoprotective switch in the major light harvesting complex II. Chem. Commun. 56, 11215–11218. 10.1039/D0CC04486E 32815976

[B18] Duffy C. D. P. Chmeliov J. Macernis M. Sulskus J. Valkunas L. Ruban A. V. (2013). Modeling of fluorescence quenching by lutein in the plant light-harvesting complex lhcii. J. Phys. Chem. B 117, 10974–10986. 10.1021/jp3110997 23234311

[B19] Fox K. F. Balevicius V. Chmeliov J. Valkunas L. Ruban A. V. Duffy C. D. P. (2017). The carotenoid pathway: what is important for excitation quenching in plant antenna complexes? Phys. Chem. Chem. Phys. 19, 22957–22968. 10.1039/C7CP03535G 28813042

[B20] Fox K. F. Ünlü C. Balevicius V. Ramdour B. N. Kern C. Pan X.. (2018). A possible molecular basis for photoprotection in the minor antenna proteins of plants. Biochim. Biophys. Acta 1859, 471–481. 10.1016/j.bbabio.2018.03.015 29625089

[B21] Fujii R. Onaka K. Kuki M. Koyama Y. Watanabe Y. (1998). The 2ag- energies of all-trans-neurosporene and spheroidene as determined by fluorescence spectroscopy. Chem. Phys. Lett. 288, 847–853. 10.1016/S0009-2614(98)00376-5

[B22] Goral T. K. Johnson M. P. Duffy C. D. P. Brain A. P. R. Ruban A. V. Mullineaux C. W. (2012). Light-harvesting antenna composition controls the macrostructure and dynamics of thylakoid membranes in arabidopsis. Plant J. 69, 289–301. 10.1111/j.1365-313X.2011.04790.x 21919982

[B23] Holleboom C.-P. Walla P. J. (2014). The back and forth of energy transfer between carotenoids and chlorophylls and its role in the regulation of light harvesting. Photosynth. Res. 119, 215–221. 10.1007/s11120-013-9815-4 23575737

[B24] Holt N. E. Zigmantas D. Valkunas L. Li X. P. Niyogi K. K. Fleming G. R. (2005). Carotenoid cation formation and the regulation of photosynthetic light harvesting. Science 307, 433–436. 10.1126/science.1105833 15662017

[B25] Horton P. Ruban A. V. Rees D. Pascal A. A. Noctor G. Young A. J. (1991). Control of the light-harvesting function of chloroplast membranes by aggregation of the lhcii chlorophyll-protein complex. FEBS Lett. 292, 1–4. 10.1016/0014-5793(91)80819-O 1959588

[B26] Horton P. Ruban A. V. Wentworth M. (2000). Allosteric regulation of the light harvesting system of photosystem ii. Philos. Trans. R. Soc. B 355, 1361–1370. 10.1098/rstb.2000.0698 11127991 PMC1692867

[B27] Ilioaia C. Johnson M. P. Liao P. N. Pascal A. A. van Grondelle R. Walla P. J.. (2011). Photoprotection in plants involves a change in lutein 1 binding domain in the major light-harvesting complex of photosystem ii. J. Biol. Chem. 286, 27247–27254. 10.1074/jbc.M111.234617 21646360 PMC3149318

[B28] Jahns P. Latowski D. Strzalka K. (2009). Mechanism and regulation of the violaxanthin cycle: the role of antenna proteins and membrane lipids. Biochim. Biophys. Acta 1787, 3–14. 10.1016/j.bbabio.2008.09.013 18976630

[B29] Johnson M. P. Goral T. K. Duffy C. D. Brain A. P. Mullineaux C. W. Ruban A. V. (2011). Photoprotective energy dissipation involves the reorganization of photosystem ii light-harvesting complexes in the grana membranes of spinach chloroplasts. Plant Cell. 23, 1468–1479. 10.1105/tpc.110.081646 21498680 PMC3101555

[B30] Johnson M. P. Ruban A. V. (2011). Restoration of rapidly reversible photoprotective energy dissipation in the absence of psbs protein by enhanced δph. J. Biol. Chem. 286, 19973–19981. 10.1074/jbc.M111.237255 21474447 PMC3103371

[B31] Khokhlov D. Belov A. (2019). Ab initio model for the chlorophyll-lutein exciton coupling in the lhcii complex. Biophys. Chem. 286, 16–24. 10.1016/j.bpc.2019.01.001 30639535

[B32] Knox R. S. Spring B. Q. (2003). Dipole strengths in the chlorophylls. Photochem. Photobiol. 77, 497–501. 10.1562/0031-8655(2003)077andlt;0497:DSITCandgt;2.0.CO;2 12812291

[B33] Krüger T. P. J. Novoderezhkin V. I. Ilioaia C. van Grondelle R. (2010). Fluorescence spectral dynamics of single lhcii trimers. Biophys. J. 98, 3093–3101. 10.1016/j.bpj.2010.03.028 20550923 PMC2884258

[B34] Lapillo M. Cignoni E. Cupellini L. Mennucci B. (2020). The energy transfer model of nonphotochemical quenching: Lessons from the minor CP29 antenna complex of plants. Biochim. Biophys. Acta 1861, 148282. 10.1016/j.bbabio.2020.148282 32721398

[B35] Li X. Gilmore A. M. Caffarri S. Bassi R. Golan T. Kramer D.. (2004). Regulation of photosynthetic light harvesting involves intrathylakoid lumen ph sensing by the psbs protein. J. Biol. Chem. 279, 22866–22874. 10.1074/jbc.M402461200 15033974

[B36] Liguori N. Campos S. R. R. Baptista A. M. Croce R. (2019). Molecular anatomy of plant photoprotective switches: The sensitivity of psbs to the environment, residue by residue. J. Phys. Chem. Lett, 10, 1737–1742. 10.1021/acs.jpclett.9b00437 30908067 PMC6477805

[B37] Liu C. Zhang Y. Cao D. He Y. Kuang T. Yang C. (2008). Structural and functional analysis of the antiparallel strands in the lumenal loop of the major light-harvesting chlorophyll a/b complex of photosystem ii (lhciib) by site-directed mutagenesis. J. Biol. Chem. 283, 487–495. 10.1074/jbc.M705736200 17959607

[B38] Liu Z. Yan H. Wang K. Kuang T. Zhang J. Gui L.. (2004). Crystal structure of spinach major light-harvesting complex at 2.72 a resolution. Nature 428, 287–292. 10.1038/nature02373 15029188

[B39] Lukeš V. Christensson N. Milota F. Kauffmann H. F. Hauer J. (2011). Electronic ground state conformers of β-carotene and their role in ultrafast spectroscopy. Chem. Phys. Lett. 506, 122–127. 10.1016/j.cplett.2011.02.060

[B40] Ma Y.-Z. Holt N. E. Li X.-P. Niyogi K. K. Fleming G. R. (2003). Evidence for direct carotenoid involvement in the regulation of photosynthetic light harvesting. Proc. Natl. Acad. Sci. U.S.A. 100, 4377–4382. 10.1073/pnas.0736959100 12676997 PMC404687

[B41] Madjet M. E. Abdurahman A. Renger T. (2006). Intermolecular coulomb couplings from ab initio electrostatic potentials: application to optical transitions of strongly coupled pigments in photosynthetic antennae and reaction centers. J. Phys. Chem. B 110, 17268–17281. 10.1021/jp0615398 16928026

[B42] Malnoë A. Schultink A. Shahrasbi S. Rumeau D. Havaux M. Niyogia K. K. (2018). The plastid lipocalin lcnp is required for sustained photoprotective energy dissipation in arabidopsis. Plant Cell. 30, 196–208. 10.1105/tpc.17.00536 29233855 PMC5810567

[B43] Malý P. Gruber J. M. van Grondelle R. Mancal T. (2016). Single molecule spectroscopy of monomeric LHCII: Experiment and theory. Sci. Rep. 6, 26230. 10.1038/srep26230 27189196 PMC4870570

[B44] Mascoli V. Liguori N. Xu P. Roy L. M. van Stokkum I. H. M. Croce R. (2019). Capturing the quenching mechanism of light-harvesting complexes of plants by zooming in on the ensemble. Chem 5, 2900–2912. 10.1016/j.chempr.2019.08.002

[B45] Müh F. Madjet M. E.-A. Renger T. (2010). Structure-based identification of energy sinks in plant light-harvesting complex II. J. Phys. Chem. B 114, 13517–13535. 10.1021/jp106323e 20886872

[B46] Müller M. G. Lambrev P. Reus M. Wientjes E. Croce R. Holzwarth A. R. (2010). Singlet energy dissipation in the photosystem II light-harvesting complex does not involve energy transfer to carotenoids. Chem. Phys. Chem. 11, 1289–1296. 10.1002/cphc.200900852 20127930

[B47] Müller P. Li X.-P. Niyogi K. K. (2001). Non-photochemical quenching. a response to excess light energy. Plant Physiol. 125, 1558–1566. 10.1104/pp.125.4.1558 11299337 PMC1539381

[B48] Nicol L. Croce R. (2021). The psbs protein and low ph are necessary and sufficient to induce quenching in the light-harvesting complex of plants lhcii. Sci. Rep. 11, 7415. 10.1038/s41598-021-86975-9 33795805 PMC8016914

[B49] Niyogi K. K. (2000). Safety valves for photosynthesis. Curr. Opin. Plant Biol. 3, 455–460. 10.1016/S1369-5266(00)00113-8 11074375

[B50] Novoderezhkin V. Marin A. Grondelle R. v. (2011). Intra- and inter-monomeric transfers in the light harvesting LHCII complex: the redfield-förster picture. Phys. Chem. Chem. Phys. 13, 17093–17103. 10.1039/c1cp21079c 21866281

[B51] Novoderezhkin V. Palacios M. A. Amerongen H. V. Grondelle R. V. (2004). Energy-transfer dynamics in the LHCII complex of higher plants: Modified redfield approach. J. Phys. Chem. B 108, 10363–10375. 10.1021/jp049600120532406

[B52] Ostroumov E. E. Götze J. P. Reus M. Lambrev P. H. Holzwarth A. R. (2020). Characterization of fluorescent chlorophyll charge-transfer states as intermediates in the excited state quenching of light-harvesting complex ii. Photosynth. Res. 144, 171–193. 10.1007/s11120-020-00745-8 32307623

[B53] Pascal A. A. Liu Z. Broess K. van Oort B. van Amerongen H. Wang C.. (2005). Molecular basis of photoprotection and control of photosynthetic light-harvesting. Nature 436, 134–137. 10.1038/nature03795 16001075

[B54] Polívka T. Sundström V. (2004). Ultrafast dynamics of carotenoid excited states-from solution to natural and artificial systems. Chem. Rev. 104, 2021–2072. 10.1021/cr020674n 15080720

[B55] Polívka T. Zigmantas D. Sundström V. Formaggio E. Cinque G. Bassi R. (2002). Carotenoid *s*_1_ state in a recombinant light-harvesting complex of photosystem ii. Bio Chem. 41, 439–450. 10.1021/bi011589x 11781082

[B56] Powles S. B. (1984). Photoinhibition of photosynthesis induced by visible light. Ann. Rev. Plant Physiol. 35, 15–44. 10.1146/annurev.pp.35.060184.000311

[B57] Renger T. Trostmann I. Theiss C. Madjet M. E. Richter M. Paulsen H.. (2007). Refinement of a structural model of a pigment-protein complex by accurate optical line shape theory and experiments. J. Phys. Chem. B 111, 10487–10501. 10.1021/jp0717241 17696386

[B58] Ruban A. V. (2016). Nonphotochemical chlorophyll fluorescence quenching: mechanism and effectiveness in protecting plants from photodamage. Plant Physiol. 170, 1903–1916. 10.1104/pp.15.01935 26864015 PMC4825125

[B59] Ruban A. V. Berera R. Ilioaia C. van Stokkum I. H. M. Kennis J. T. M. Pascal A. A.. (2007). Identification of a mechanism of photoprotective energy dissipation in higher plants. Nature 450, 575–578. 10.1038/nature06262 18033302

[B60] Ruban A. V. Horton P. (1999). The xanthophyll cycle modulates the kinetics of nonphotochemical energy dissipation in isolated light-harvesting complexes, intact chloroplasts, and leaves of spinach. Plant Physiol. 119, 531–542. 10.1104/pp.119.2.531 9952449 PMC32130

[B61] Ruban A. V. Johnson M. P. Duffy C. D. P. (2012). The photoprotective molecular switch in the photosystem ii antenna. Biochim. Biophys. Acta 1817, 167–181. 10.1016/j.bbabio.2011.04.007 21569757

[B62] Ruban A. V. Wilson S. (2020). The mechanism of non-photochemical quenching in plants: localisation and driving forces. Plant Cell Physiol. 62, 1063–1072. 10.1093/pcp/pcaa155 33351147

[B63] Saccon F. Durchan M. Bína D. Duffy C. D. Ruban A. V. Polívka T. (2020). A protein environment-modulated energy dissipation channel in lhcii antenna complex. iScience 23, 101430. 10.1016/j.isci.2020.101430 32818906 PMC7452274

[B64] Sato R. Ohta H. Masuda S. (2014). Prediction of respective contribution of linear electron flow and pgr5-dependent cyclic electron flow to non-photochemical quenching induction. Plant Physiol. Bio Chem. 81, 190–196. 10.1016/j.plaphy.2014.03.017 24725611

[B65] Son M. Pinnola A. Bassi R. Schlau-Cohen G. S. (2019). The electronic structure of lutein 2 is optimized for light harvesting in plants. Chem 5, 575–584. 10.1016/j.chempr.2018.12.016

[B66] Son M. Pinnola A. Gordon S. C. Bassi R. Schlau-Cohen G. S. (2020). Observation of dissipative chlorophyll-to-carotenoid energy transfer in light-harvesting complex II in membrane nanodiscs. Nat. Commun. 11, 1295. 10.1038/s41467-020-15074-6 32157079 PMC7064482

[B67] Staleva H. Komenda J. Shukla M. K. Šlouf V. Kaňa R. Polívka T.. (2015). Mechanism of photoprotection in the cyanobacterial ancestor of plant antenna proteins. Nat. Chem. Biol. 11, 287–291. 10.1038/nchembio.1755 25706339

[B68] Strand D. D. Kramer D. M. (2014). Control of non-photochemical exciton quenching by the proton circuit of photosynthesis, in Non-Photochemical Quenching and Energy Dissipation in Plants, Algae and Cyanobacteria, eds. Demmig-Adams B. G. Garab W. A. III Govindjee U. (Dordrecht: Springer), 387–408. 10.1007/978-94-017-9032-1_18

[B69] Tavan P. Schulten K. (1987). Electronic excitations in finite and infinite polyenes. Phys. Rev. B 36, 4337. 10.1103/PhysRevB.36.4337 9943414

[B70] Walla P. J. Linden P. A. Ohta K. Fleming G. R. (2002). Excited-state kinetics of the carotenoid s1 state in lhc ii and two-photon excitation spectra of lutein and β-carotene in solution: efficient car s1-chl electronic energy transfer via hot s1 states? J. Phys. Chem. A 106, 1909–1916. 10.1021/jp011495x

[B71] Walla P. J. Yom J. Krueger B. Fleming G. (2000). Two photon excitation spectrum of lhcii and fluorescence up-conversion after one- and two-photon excitation of the carotenoids. J. Phys. Chem. B 104, 4799–4806. 10.1021/jp9943023

[B72] Walters R. G. Ruban A. V. Horton P. (1994). Higher plant light-harvesting complexes lhciia and lhciic are bound by dicyclohexylcarbodiimide during inhibition of energy dissipation. Eur. J. Bio Chem. 226, 1063–1069. 10.1111/j.1432-1033.1994.01063.x 7813461

[B73] Wei T. Balevicius V. Polívka T. Ruban A. V. Duffy C. D. P. (2019). How carotenoid distortions may determine optical properties: lessons from the orange carotenoid protein. Phys. Chem. Chem. Phys. 21, 23187–23197. 10.1039/C9CP03574E 31612872

[B74] Wei X. Su X. Cao P. Liu X. Chang W. Li M.. (2016). Structure of spinach photosystem II-LHCII supercomplex at 3.2 åresolution. Nature 534, 69–74. 10.1038/nature18020 27251276

[B75] Xu P. Tian L. Kloz M. Crocea R. (2015). Molecular insights into zeaxanthin-dependent quenching in higher plants. Sci. Rep. 5, 13679. 10.1038/srep13679 26323786 PMC4555179

