# Supplementary material for: Trivial Excitation Energy Transfer to Carotenoids Is an Unlikely Mechanism for Non-photochemical Quenching in LHCII
Source: Front Plant Sci. 2022 Jan 13;12:797373. doi: 10.3389/fpls.2021.797373 (PMC8792765; doi:10.3389/fpls.2021.797373)
Supplement: Supplementary file 1 [file Data_Sheet_1.pdf]

1 VERA parameters for lutein

$\omega_{S_0}, \omega_{S_1}, \omega_{S_2}, \omega_{S_n}$	0 cm^{-1}, 14,050 cm^{-1}, 20300cm^{-1}, 31950cm^{-1}
$\omega_{C\text{-}C}, \omega_{C=C}$	1156cm^{-1}, 1523 cm^{-1}
$\lambda_{S_0}, \lambda_{S_1}, \lambda_{S_2}$	15cm^{-1}, 100 cm^{-1}, 150cm^{-1}
$\lambda_{S_0-S_1}$	31cm^{-1}
$\lambda_{S_1-S_2}$	860cm^{-1}
γ_i, γ_{ij}	163.6 fs
$d^{S_0-S_1}_{C\text{-}C}, d^{S_0-S_1}_{C=C}$	0.82, 0.82
$d^{S_0-S_2}_{C\text{-}C}, d^{S_0-S_2}_{C=C}$	0.70, 0.84
$d^{S_1-S_2}_{C\text{-}C}, d^{S_1-S_2}_{C=C}$	0.80, 0.80
$d^{S_1-S_n}_{C\text{-}C}, d^{S_1-S_n}_{C=C}$	0.55, 0.0
$\Delta\omega_{S_0-S_1}, \Delta\omega_{S_0-S_2}, \Delta\omega_{S_1-S_n}$	1070cm^{-1}, 1190cm^{-1}, 1090cm^{-1}
$\frac{\lvert\mu_{S_1-S_n}\rvert^2}{\lvert\mu_{S_0-S_2}\rvert^2}$	1.22
S_2 Stokes shift	150cm^{-1}

Table 1: The total parameter set for out vibronic model of Lut in pyridine, as described in the main text

2 Secular Redfield model of the Chl manifold

As outlined briefly in the main text we use secular Redfield theory for the modelling of energy relaxation on Chl exciton manifold. The starting point is the spectral density of energy gap fluctuations for the uncoupled Chls for which we assume the ansatz spectral density proposed by Novoderezhkin et al [1].

$$C''_n(\omega) = \sum_{j=1}^{N=48} 2S_j\omega_j \frac{\omega\omega_j^2\gamma_j}{(\omega^2 - \omega_j^2)^2 + \omega^2\gamma_j^2} + 2\lambda_0 \frac{\omega\gamma_0}{\omega^2 + \gamma_0^2} \tag{1}$$

Here ω_i are the frequencies of the 48 under-damped modes, S_i are the associated Huang-Rhys factors and γ_i are the damping times. The over-damped (Drude) term is characterized by is own damping time, γ_0, and a

reorganization energy λ_0. The effective reorganisation energy is given by

$$\lambda_n = \frac{1}{\pi}\int_0^{\infty} \frac{C''_n(\omega)}{\omega} d\omega \tag{2}$$

and the energy-gap correlation function (in the frequency domain) is given by the fluctuation-dissipation theorem,

$$C_n(\omega) = \left(1 + \coth\left(\frac{\hbar\omega}{k_B T}\right)\right) C''_n(\omega) \tag{3}$$

Switching to the exciton basis, the transition dipole moments, reorganization energies, relaxation rates and correlation functions (in the time domain) mix according to,

$$\mu_i(t_k) = \sum_n c_n^i(t_k)\mu_n(t_k) \tag{4}$$

$$\lambda_i(t_k) = \sum_n \left|c_n^i(t_k)\right|^4 \lambda_n \tag{5}$$

$$\Gamma_i = \sum_n \left|c_n^i(t_k)\right|^2 \Gamma_n \tag{6}$$

$$C_i(t;t_k) = \sum_n \left|c_n^i(t_k)\right|^2 C_n(t) \tag{7}$$

with the participation coefficients $\{c_n^i(t_k)\}$ and uncoupled (site) transition dipoles, $\mu_n(t_k)$ varying from snapshot to snapshot. Γ_n^{-1} are the excitation lifetimes of the uncoupled Chls which are all assinged the typical experimental value of $4ns$. The exciton line-broadening functions are expressed in terms of $C''_i(\omega;t_k)$

$$g_{ii}(t;t_k) = \frac{1}{\pi}\int_0^{\infty} d\omega \frac{C''_i(\omega;t_k)}{\omega^2}\left[\coth\left(\frac{\hbar\omega}{2k_b T}\right)(1-\cos(\omega t)) + i\left(\sin(\omega t) - \omega t\right)\right] \tag{8}$$

which in turn gives the instantaneous (snapshot) exciton LA,

$$\chi_i(\omega;t_k) \propto |\mu_i(t_k)|^2 \Re \int_0^{\infty} d\tau \exp\left[-i(\omega - \omega_i(t_k))\tau - g_{ii}(\tau;t_k) - \frac{\Gamma_i(t_k)}{2}\tau\right] \tag{9}$$

and FL,

$$\widetilde{\chi_i}(\omega;t_k) \propto |\mu_i|^2 \Re \int_0^{\infty} d\tau \exp\left[-i(\omega - \omega_i(t_k) + 2\lambda_i(t_k))\tau - g^*_{ii}(\tau;t_k) - \frac{\Gamma_i(t_k)}{2}\tau\right] \tag{10}$$

line-shapes respectively. These are used to calculate the LA and FL spectra as in the main text. Finally, the population dynamics, $\{P_i(t;t_k)\}$, of a single

MD snap-shot are given by a set of Master Equations,

$$\frac{dP_i(t;t_k)}{dt} = -\left(\sum_{j\neq i} k_{j\leftarrow i}(t_k) + \Gamma_i(t_k)\right) P_i(t;t_k) + \sum_{j\neq i} k_{i\leftarrow j}(t_k) P_j(t;t_k) \quad (11)$$

with the rate constants defined in the main text. Note we completely ignore the coherences as they are not relevant to the timescales being probed.

3 The Vibrational Energy Relaxation Approach (VERA) to the Lut dynamics interaction

We use the VERA approach developed by Balevičius et al. [2] the basic assumptions of which are discussed in the main text. The time-evolution of the vibronic populations are given by,

$$\frac{dn^i_{a_1a_2}}{dt} = \left(\frac{dn^i_{a_1a_2}}{dt}\right)_{IVR} + \left(\frac{dn^i_{a_1a_2}}{dt}\right)_{IC} + \left(\frac{dn^i_{a_1a_2}}{dt}\right)_{pump} \quad (12)$$

where the three terms correspond to the vibrational relaxation on the electronic states (IVR), interconversion (IC) between electronic states, and the initial resonant excitation by the pump pulse. The IVR is determined by

$$\left(\frac{dn^i_{a_1a_2}}{dt}\right)_{IVR} = -\left(a_1k_1^- + a_2k_2^- + (a_1+1)k_1^+ + (a_2+1)k_2^+\right) n^i_{a_1a_2} \quad (13)$$

$$+ (a_1+1)k_1^- n^i_{a_1+1a_2} + (a_2+1)k_2^- n^i_{a_1a_2+1} \quad (14)$$

$$+ a_1k_1^+ n^i_{a_1-1a_2} + a_2k_2^+ n^i_{a_1a_2-1} \quad (15)$$

where the first line denotes loss of population to upper and lower vibrational states on mode 1 and 2 and the second set of four terms denote incoming population from those states. We define upward, k^+, and downward, k^-, vibrational relaxation rates as

$$k^\pm_\alpha = C''_c(\mp|\omega_\alpha|)\left[\coth\left(\mp\frac{\beta\hbar|\omega_\alpha|}{2}\right) + 1\right] \quad (16)$$

Note that in the harmonic approximation we neglect overtone ($\Delta a_\alpha > a_\alpha \pm 1$) transitions or couplings between the two modes. For the IC dynamics we have

$$\left(\frac{dn^i_{a_1a_2}}{dt}\right)_{IC} = \sum_{b_1,b_2}\left(\prod_{\alpha=1,2}\left|F^\alpha_{i_{a_\alpha},i+1_{b_\alpha}}\right|^2\right)\left[-k^{i+1,i}_{b_1,b_2,a_1,a_2} n^i_{a_1a_2} + k^{i,i+1}_{a_1a_2,b_1b_2} n^{i+1}_{b_1b_2}\right] \quad (17)$$

$$+ \sum_{b_1,b_2}\left(\prod_{\alpha=1,2}\left|F^\alpha_{i_{a_\alpha},i+1_{b_\alpha}}\right|^2\right)\left[-k^{i-1,i}_{b_1,b_2,a_1,a_2} n^i_{a_1a_2} + k^{i,i-1}_{a_1a_2,b_1b_2} n^{i-1}_{b_1b_2}\right] \quad (18)$$

where the first pair of terms describe the upward and downward transitions between $|i_{a_1a_2}\rangle \leftrightarrow |i+1_{b_1b_2}\rangle$ respectively and the second pair describe the upward and downward transitions between $|i_{a_1a_2}\rangle \leftrightarrow |i-1_{b_1b_2}\rangle$ respectively. Here the rate constants $k^{ij}_{a_1a_2,b_1b_2}$ are defined as

$$k^{i>j}_{a_1a_2,b_1b_2} = C''_f\left(-\Delta^{ij}_{a_1a_2,b_1b_2}\right)\left[\coth\left(-\frac{\beta\hbar\Delta^{ij}_{a_1a_2,b_1b_2}}{2}\right)+1\right] \tag{19}$$

$$k^{i<j}_{a_1a_2,b_1b_2} = C''_f\left(\Delta^{ji}_{a_1a_2,b_1b_2}\right)\left[\coth\left(\frac{\beta\hbar\Delta^{ji}_{a_1a_2,b_1b_2}}{2}\right)+1\right] \tag{20}$$

with $\Delta^{ij}_{a_1a_2,b_1b_2}$ defined as in the main text.
